# Very severe aplastic anemia in a child with pulmonary mucormycosis: a case report

**DOI:** 10.3389/fped.2024.1453059

**Published:** 2024-09-12

**Authors:** Minchun Huang, Nanchuan Jiang, Yahui Ren, Yun Peng, Xiaoyan Wu

**Affiliations:** ^1^ Department of Pediatrics, Union Hospital, Tongji Medical College, Huazhong University of Science and Technology, Wuhan, China; ^2^ Department of Radiology, Union Hospital, Tongji Medical College, Huazhong University of Science and Technology, Wuhan, China

**Keywords:** pulmonary mucormycosis, left lower lobectomy, metagenomic next-generation sequencing, reverse halo sign, very severe aplastic anemia

## Abstract

Aplastic anemia (AA), is a rare but potentially life-threatening disease characterized by pancytopenia and a hypocellular bone marrow. Pulmonary mucormycosis (PM) is a rare but life-threatening fungal infection observed in immunocompromised patients, particularly those with neutropenia and those using corticosteroids, with a high mortality rate from 40 to 80%. However, PM diagnosis and treatment remain challenging. This study reports a case of very severe aplastic anemia (VSAA) in a male child with PM. The innovation of this article lies in the following aspects: the patient exhibited typical clinical manifestations, the reverse halo sign (RHS) on chest computed tomography (CT), and a positive metagenomic next-generation sequencing (mNGS) analysis; despite aggressive anti-infective treatment and left lower lobectomy, he experienced a poor clinical outcome. Reflecting on cases with poor prognosis can indeed offer valuable insights and opportunities for learning. This study underlines the diagnostic challenges in mucormycosis, which should be considered in persistent fever that is unresponsive to standard antibiotic and antifungal therapies, and conduct a comprehensive examination to achieve early detection, diagnosis and treatment. It was concluded that, in addition to antifungal treatment, early surgery is essential for treating mucormycosis.

## 1 Introduction

Aplastic anemia (AA), a rare but potentially life-threatening disease, is a paradigm of bone marrow failure syndromes characterized by the loss of hematopoietic stem cell, bone marrow failure, and peripheral pancytopenia (1, 2). Mucormycosis is a fungal disease that mainly infects immunocompromised patients, including those with uncontrolled diabetes mellitus, hematologic malignancies stem cell transplants, neutropenia, and corticosteroid usage, and can be life-threatening (3, 4). Invasive mucormycosis can involve many different organs, predominantly the rhinocerebral region, followed by pulmonary involvement (3, 5, 6). Severe neutropenic patients, typically manifest pulmonary mucormycosis (PM), whereas those with diabetic individuals often have rhino-orbital disease. PM is recognized as an aggressive fungal disease with a high mortality rate from 40 to 80% (4). Early diagnosis and treatment of PM is challenging because of the absence of specific clinical manifestations, which causes clinical outcomes despite aggressive therapeutic interventions. While extensive literature has been reviewed, only a limited number of PM cases associated with very severe aplastic anemia (VSAA) have been studied. This article reports a typical case of PM associated with rapid progression on chest computed tomography (CT), with the etiology detected by metagenomic next-generation sequencing (mNGS), caused by Cunninghamella bertholletiae in a child with VSAA. This case typically provides potential insights into the diagnosis and treatment of PM patients.

## 2 Case description

**Figure 1:**
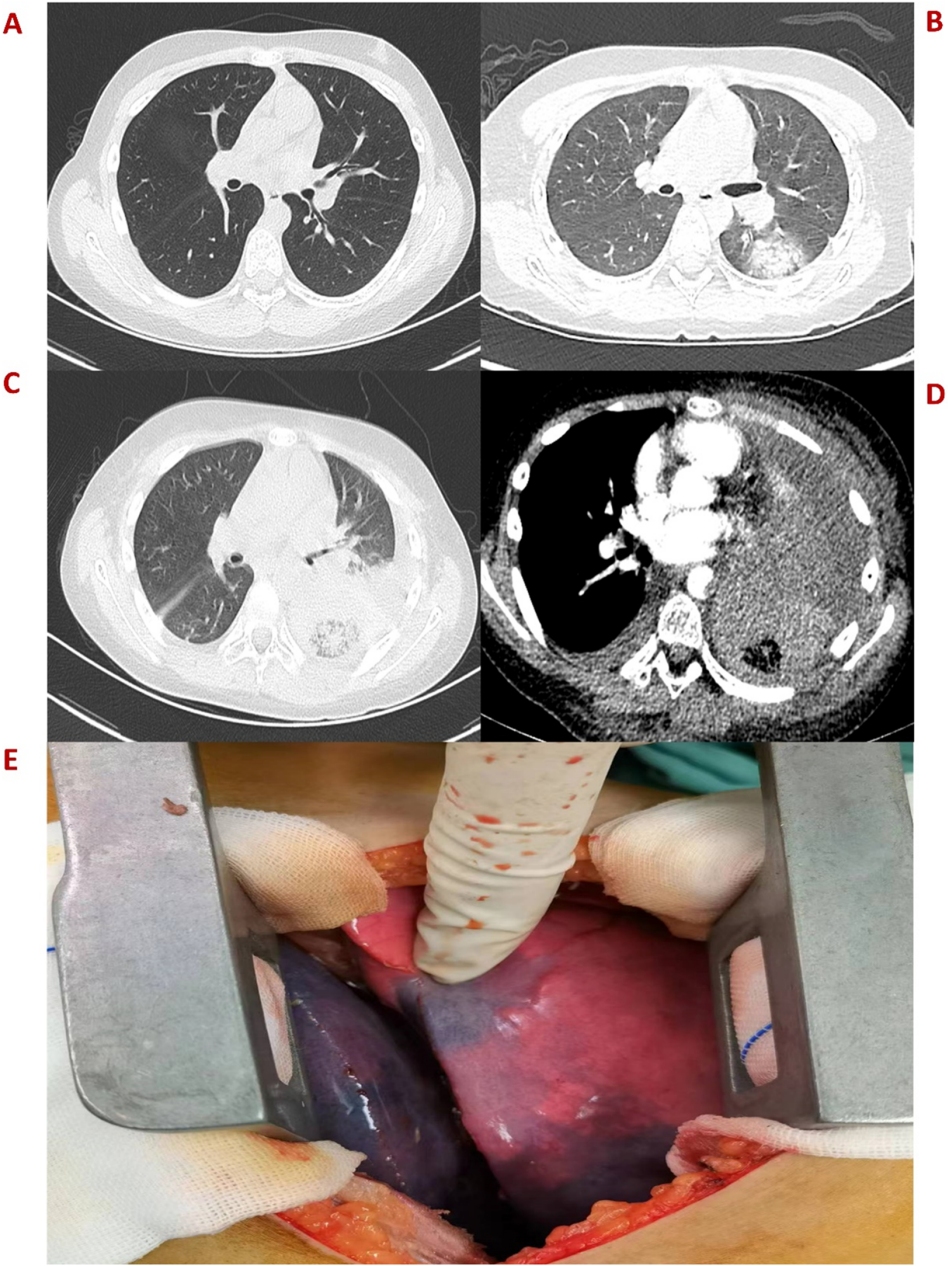
**(A)** Plain CT scan image of the left lung on day 2. **(B)** An emergency CT scan image obtained after 12 days indicates patchy ground-glass shadows in the dorsal segment of the left lower lobe. **(C)** An enhanced CT image obtained after 22 days shows a reverse halo sign in the left lung. **(D)** Enhanced CT image obtained on day 30 presents the left lung infarction. **(E)** Surgical photos of lesions showing the left lower lobe infarction.

**Figure 2:**
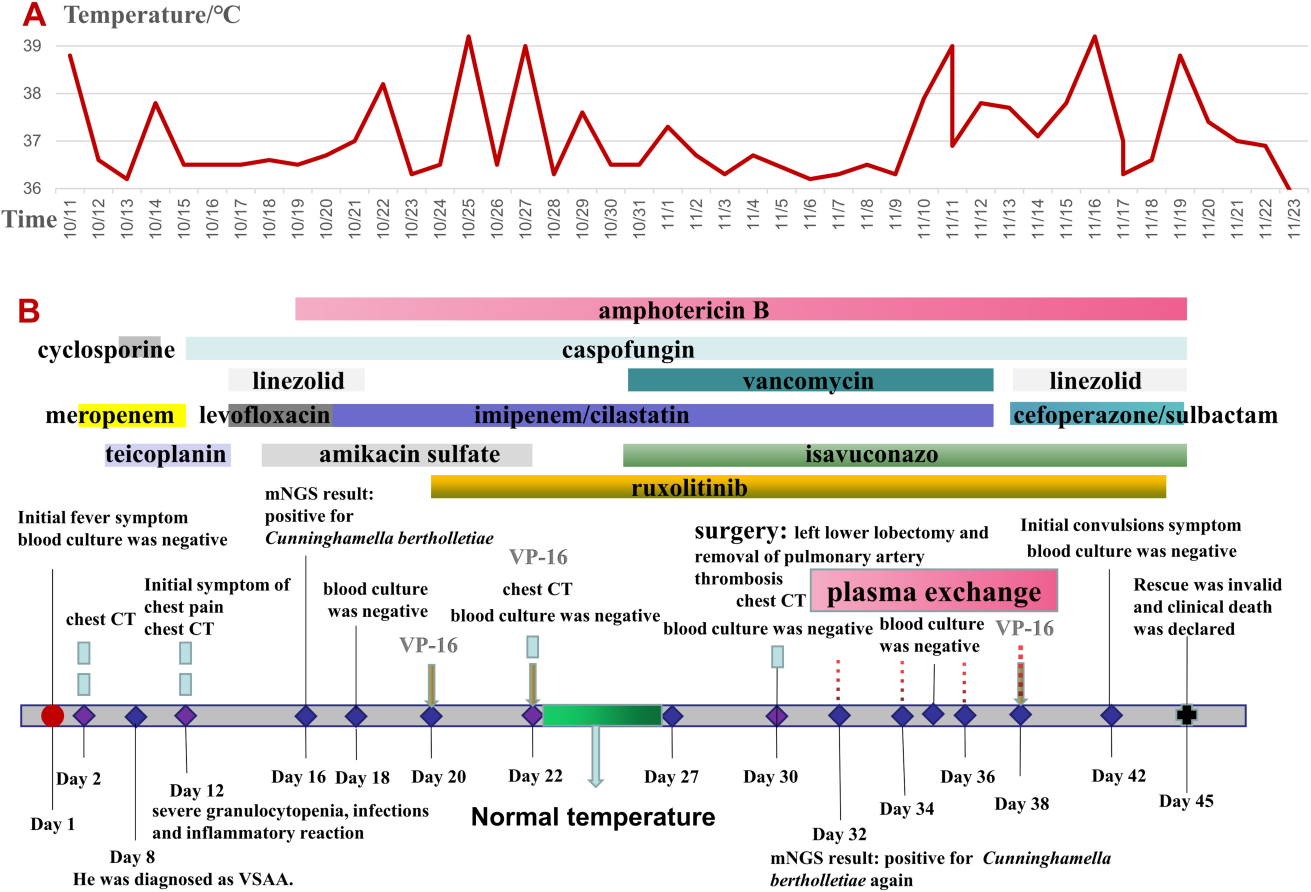
**(A)** Core body temperature curves. **(B)** The treatment timeline. VSAA, very severe aplastic anemia; CT, computed tomography; VP – 16, etoposide; mNGS, metagenomic next-generation sequencing.

An 11-year-old boy was admitted to the hospital on October 11, 2022, due to hemorrhagic spots on the skin that had lasted for 1 week, and gingival bleeding that had been present for 1 day. He came from a well-off family and there was no previous medical, family, and psychosocial history. Initial laboratory analysis revealed the following data: white blood cell count 0.97 × 10^9^/L, absolute neutrophil count 0.06 × 10^9^/L, hemoglobin count 99 g/L, reticulocyte count 0.01 × 10^9^/L, platelet count 1 × 10^9^/L. The blood test showed a simultaneous decrease in the ternary system. The nucleated cell count in bone marrow was severely reduced, particularly because of leukocytes (92.5%) and not megakaryocytes. The bone marrow pathological analysis revealed a notable reduction of hematopoietic cells and an increase in non-hematopoietic cells such as fat cells. Moreover, his full exon sequencing, chromosomal aberration, comet, and gene mutation assays were negative. AA is a hematopoietic stem cell disorder featuring reduced bone marrow cellularity and decreased hematopoiesis. The diagnosis of aplastic anemia requires the presence of all four of these criteria: (1) Peripheral blood cytopenia, defined as two or more of the following: hemoglobin <100 g/L, absolute neutrophil count < 1.5 × 10^9^/L, platelet count < 50 × 10^9^/L. (2) Hypocellular bone marrow, defined as the presence of a bone marrow biopsy with cellularity <25% of normal for the patient's age. (3) Absence of dysplasia, infiltration, or fibrosis in the bone marrow. (4) Exclusion of other causes of pancytopenia, such as paroxysmal nocturnal hemoglobinuria, myelodysplastic syndrome, or other hematological malignancies. The modified Camitta criteria classified AA into three categories on the basis of bone marrow cellularity, an absolute neutrophil count, platelet count, and reticulocyte count. The criteria of SAA included marrow hypocellularity < 25% and at least two of the following: absolute neutrophil count < 0.5 × 10^9^/L, platelet count < 20 × 10^9^/L, and reticulocyte count < 20 × 10^9^/L. Additionally, in VSAA, there is extreme neutropenia of < 0.2 × 10^9^/L (1, 7). Based on Camitta criteria, the child was diagnosed with VSAA. Although there are many known etiologies; however, unlike the majority of the cases, the etiology of VSAA was difficult to determine in this child (1, 2), and he was managed as a case of idiopathic acquired VSAA. To treat the primary disease, he was given cyclosporine on day 8 until a rash appeared on day 10. After the admission, the child developed a fever with no obvious swelling of the liver, spleen, or lymph nodes. Laboratory analysis revealed the following data: white blood cell count 0.58 × 10^9^/L, absolute neutrophil count 0.08 × 10^9^/L, hemoglobin count 83 g/L, reticulocyte count 0.01 × 10^9^/L, platelet count 1 × 10^9^/L, C-reactive protein (CRP) count 52 mg/L, and ferritin count 450 ng/dl. Furthermore, 1, 3-b-D glucan, serum galactomannan antigen, and blood culture analyses indicated negative results on multiple occasions. All pathogen tests including epstein-barr virus virus, cytomegalo virus, hepatitis virus, etc.,are negative, and no obvious infectious lesions are found in the CT scans of the head and chest (Figure 1A) and in the abdominal ultrasound. On the day of admission with neutropenia and fever, meropenem was added immediately for anti-infective treatment. Despite aggressive anti-infection therapy (i.e., meropenem, teicoplanin, linezolid, levofloxacin, and imipenem/cystatin) (Figure 2) for nearly two weeks, there was persistent fever, intermittent hemoptysis, and chest wall pain. These persistent clinical symptoms and rapid progression of pulmonary infection as evident in the pulmonary CT (indicated an area of ground-glass opacity surrounded by consolidation on day 12 (Figure 1B), led to the diagnosis of a mixed bacterial and fungal infection. Antibiotics were changed and antifungal drug (caspofungin) was added as the symptoms of infection were not alleviated. To treat severe granulocytopenia, infections, and inflammatory response, on day 12 the granulocyte colony-stimulating factor (G-CSF), gammaglobulin, and methylprednisolone were administered. On the day 16th, mNGS indicated that he was positive for Cunninghamella bertholletiae. As mNGS suggested a single infection of Cunninghamella bertholletiae, amphotericin B was added for antifungal treatment immediately. In the chest CT on day 22, the left lung revealed a consolidation enlargement with central ground-glass shadows, recognized as the reverse halo sign (RHS) (Figure 1C), indicative of a characteristic feature of PM (6). Although the antifungal therapy (i.e., amphotericin B liposome, caspofungin, and isavuconazole) (Figure 2) was started, there was still a persistent fever, severe agranulocytosis, and uncontrolled pulmonary infection. Notably, the lung CT suggested the presence of a left lower lobe infarction on day 30 (Figure 1D). Therefore, as a timely intervention, emergency surgery was performed, which caused a left lower lobe infarction during the procedure (Figure 1E), confirming the initial suspicion. After collaboration with the radiology department, a pulmonary infarction was identified. Subsequently, a timely surgical intervention involving a left lower lobectomy and the removal of pulmonary artery thrombosis was performed, which effectively preserved the crucial time for the patient's survival. During the surgery, routine histopathological examination of the lung tissue was performed, but the result was negative. Despite the successful operation and the administration of amphotericin B into the chest cavity via the left thoracic drainage tube, the patient continued to suffer from a severe infection. This persistent infection stimulated an increased inflammatory response, as evident by progressively elevated levels of CRP (>200 mg/L) and ferritin (>10,000 ng/dl). Although the fungal culture test was negative, mNGS detection performed on pleural effusion revealed the presence of Cunninghamella bertholletiae on day 32, indicating a severe infection and elevated inflammatory response. Multiple postoperative chest x-ray examinations revealed left pleural effusion and a progressive worsening of pulmonary infection. Based on adequate anti-infective treatment, methylprednisolone, etoposide, ruxolitinib, and plasma exchange were administered to control inflammatory response (Figure 2). On day 42, the child went into convulsions, indicating a deteriorating condition. Since initially, the cranial CT appeared normal, intracranial infection was diagnosed. Subsequent lumbar puncture revealed clear yellow cerebrospinal fluid (CSF) with significantly low glucose, elevated protein, and decreased chloride levels, confirming an intracranial infection. Consequently, intensified anti-infective therapy was initiated. Despite the administration of intensive therapies, he died of severe septic shock and disseminated intravascular coagulation dysfunction (DIC) on day 45. The Ethics Committee of the Union Hospital of Tongji Medical College, Huazhong University of Science and Technology approved this study.

## 3 Discussion

AA is an uncommon bone marrow disorder. Laboratory and clinical observations suggest an aberrant immune response, leading to the activation of type 1 cytotoxic T cells, which destroy hematopoietic stem cell progenitors (8).The estimated incidence of AA is around 1–2 cases per million per year, which is about three times higher in East Asia. Survival rates at 3 months, and at 2 and 15 years after the diagnosis were 73%, 57%, and 51%, respectively (9). Mucormycosis is a rare but aggressive infection caused by fungi of the order Mucorales and has a high mortality rate ranging from 40 to 80% (4). This condition is common in immunocompromised patients, especially in those with uncontrolled diabetes mellitus, hematologic malignancy stem cell transplant, neutropenia, and those who use corticosteroids (3, 6, 10). Cunninghamella bertholletiae is an unusual opportunistic pathogen classified under the class Zycomycetes Fungi (11, 12) and is a rare cause of mucormycosis, predominantly affecting immunosuppressed hosts (13). Neutropenia is an independent risk factor for PM (5, 13). Most reported patients diagnosed with VSAA have indicated severe neutropenia and corticosteroid use, which are important risk factors for PM. Despite the scarcity of reported PM cases in VSAA patients, it remains a noteworthy cause of mortality. The clinical manifestation of PM typically lacks specificity and presents the following symptoms: cough, fever, chest pain, and hemoptysis, depending on the immune status of the host (5). A study on 92 PM patients indicated hemoptysis in 28.3% of cases. Moreover, severe hemoptysis is considered specifically associated with PM (14) and because it invades blood vessels, it can cause rapid and diffuse infection, causing death in some patients within a short time (15). Therefore, if a patient is diagnosed with PM and experiences hemoptysis, early initiation of surgical intervention and antifungal treatment is imperative to maximize the chances patient's survival.

Unfortunately, the laboratory test results are negative, which is almost indistinguishable from other more common molds (14). The RHS manifests as an area of consolidation with central ground-glass shadows on pulmonary imaging and was initially described in patients with cryptogenic organizing pneumonia, and has been identified as a specific marker of PM (6). Legouge et al. reported that CT examinations indicated that RHS was present in 94% of PM patients, particularly in the early stages, and diminished in subsequent stages. Hence, the chest CT should be performed in time. In diseases that are challenging to differentiate, a comprehensive approach that integrates clinical context is essential. Here, based on the patient's symptoms and the relatively rapid progression observed on chest CT, PM was diagnosed, which was further corroborated by mNGS. In the present case, Cunninghamella bertholletiae was detected via mNGS on days 16 and 32, while the fungal cultures were consistently negative. This highlights the potential of mNGS in facilitating the diagnosis of mucormycosis. Although mucormycosis is acknowledged as an opportunistic pathogen, the persistent fever, rapid progression observed on the chest CT, and the ineffectiveness of antibiotic treatment collectively confirmed PM diagnosis. At present, the diagnosis of PM depends on microbial culture or pathology (4, 16). However, blood cultures rarely cultivate pathogenic bacteria despite the angio-invasive nature of mucormycosis. The gold standard diagnostic technique remains the pathologic findings of a tissue biopsy (16). Nonetheless, this invasive procedure isn't universally accepted by patients, adding complexity to PM diagnosis, and significantly impacting clinical treatment and prognosis. Because of the high morbidity and mortality rates of PM, early diagnosis and intervention are crucial for improving survival rates. A multidisciplinary team comprising medical, surgical, radiology, and laboratory-based expertise is essential (4). The combination of medical and surgical approaches enhances the survival rates more than the medical treatment alone (14). Mortality rates of PM after medical treatment alone range from approximately 35%–100%, whereas it was 0%–50% after combining complete surgical debridement and medical treatment (17). Some literature advocates the use of a lipid formulation of amphotericin B as first-line therapy for mucormycosis (4). Here, the patient was administered amphotericin B upon suspicion of Cunninghamella bertholletiae infection. Because of its invasiveness of blood vessels, PM patients with severe agranulocytosis could progress much faster than patients with non-malignant hematological diseases. PM remains a disease with a high mortality rate and despite active treatment, the patient reported, still died of severe septic shock.

The adverse results of the current PM case revealed several clinical implications that should be considered: (1) The anti-PM treatment failed because of uncontrolled hematological diseases. (2) While adjusting the anti-infective treatment, fiberoptic bronchoscopy alveolar lavage should be performed and bronchoalveolar lavage fluid (BALF) should be sent for mNGS testing. (3) In case of severe agranulocytosis, antifungal therapy should be administered as soon as possible if the anti-bacterial infection therapy is ineffective. (4) Despite successive administration of anti-infective treatment including methylprednisolone, etoposide, ruxolitinib, and plasma exchange to control inflammatory reactions, the patient still experienced a severe fungal infection and inflammatory response. This underscores the need for further studies to optimize immunosuppression strategies. (5) In cases where chest CT indicates rapid lesion progression and ineffective medical treatment, early surgical intervention should be contemplated. Timely surgical measures can potentially increase the chances of survival that could otherwise be fatal.

## 4 Conclusion

This study reported a case of a VSAA patient with PM, who was treated with antifungal therapies and left lower lobectomy because of left pulmonary infarction. Despite aggressive treatment strategies, the patient's clinical outcome remained unfavorable, highlighting the challenges associated with PM management. Reflecting on cases with poor prognosis can indeed offer valuable insights and opportunities for learning. Therefore, using multiple analysis methods for diagnosing PM is emphasized. Though the gold standard for diagnosis of PM still depends on pathological tissue biopsy, RHS can be identified by chest CT in the early stage. mNGS also can facilitate the diagnosis of mucormycosis by providing clinical and therapeutic information, in support of conventional diagnostic approaches. Moreover, the patients should be continuously monitored for lung infarction when suffering chest pain. In addition to antifungal treatment, early surgical intervention is essential for treating mucormycosis, which can improve the survival rate.

## Data Availability

The original contributions presented in the study are included in the article/Supplementary Material, further inquiries can be directed to the corresponding author.
